# Exceptionally Preserved Jellyfishes from the Middle Cambrian

**DOI:** 10.1371/journal.pone.0001121

**Published:** 2007-10-31

**Authors:** Paulyn Cartwright, Susan L. Halgedahl, Jonathan R. Hendricks, Richard D. Jarrard, Antonio C. Marques, Allen G. Collins, Bruce S. Lieberman

**Affiliations:** 1 Department of Ecology and Evolutionary Biology, University of Kansas, Lawrence, Kansas, United States of America; 2 Department of Geology and Geophysics, University of Utah, Salt Lake City, Utah, United States of America; 3 Department of Geology, and Division of Invertebrate Paleontology, Natural History Museum, University of Kansas, Lawrence, Kansas, United States of America; 4 Departamento de Zoologia, Instituto de Biociências, Universidade de São Paulo, São Paulo, São Paulo, Brazil; 5 National Systematics Laboratory of NOAA Fisheries Service, National Museum of Natural History, Smithsonian Institution, Washington, D. C., United States of America; University of Sheffield, United Kingdom

## Abstract

Cnidarians represent an early diverging animal group and thus insight into their origin and diversification is key to understanding metazoan evolution. Further, cnidarian jellyfish comprise an important component of modern marine planktonic ecosystems. Here we report on exceptionally preserved cnidarian jellyfish fossils from the Middle Cambrian (∼505 million years old) Marjum Formation of Utah. These are the first described Cambrian jellyfish fossils to display exquisite preservation of soft part anatomy including detailed features of structures interpreted as trailing tentacles and subumbrellar and exumbrellar surfaces. If the interpretation of these preserved characters is correct, their presence is diagnostic of modern jellyfish taxa. These new discoveries may provide insight into the scope of cnidarian diversity shortly after the Cambrian radiation, and would reinforce the notion that important taxonomic components of the modern planktonic realm were in place by the Cambrian period.

## Introduction

The phylum Cnidaria is one of the earliest branching animal groups to display organized tissues and a nervous system [1]. Cnidaria has a primary divergence between two large clades, Anthozoa, corals and sea anemones, and Medusozoa, including scyphozoans (true jellyfish), cubozoans (box jellies), hydrozoans (hydroids, *Hydra*, and hydromedusae) and staurozoans (stalked medusae) [2]. Typically, medusozoan cnidarians have a pelagic, predatory jellyfish stage in their life cycle; staurozoans are the exceptions. The predatory life-style is accompanied by sense organs and sophisticated behavior, although little is known about the evolution of these complex traits. Because of their early origin within Metazoa, genomic and developmental studies of cnidarians are yielding important insights into the unfolding of animal diversity, e.g., [3]–[5]. The early divergence of cnidarians in animal phylogeny leaves little doubt of their presence in the Cambrian; however, there have been no previous reports of fossils possessing preserved characters diagnostic of particular medusozoan clades. The absence of preservation detail in medusozoan fossils has thus far hampered our knowledge of the extent of cnidarian diversity and complexity that existed during this key time in animal evolution.

Many fossils of soft-bodied medusoid-like animals have been reported from the late Neoproterozoic and Cambrian, but the lack of diagnostic characters obscures their phylogenetic provenance [6]. Fossil chondrophores (family Porpitidae) are the only possible evidence for crown group hydrozoans during the Late Proterozoic and Cambrian [6]–[11] but see [12]. Porpitids, however, are not true medusae, and instead are pelagic colonies whose individual members are organized around a large central mouth. Many previously described non-porpitid medusoid-like forms from the Ediacaran and Cambrian are preserved as circular impressions in siliciclastic or calcareous sediments thought to have been deposited in shallow marine conditions [13], [14]. Typically, these fossils are preserved in low relief and often display concentric internal rings that are sometimes overlapped by radiating lines. Most of these medusoid-like fossils lack distinctive evidence of soft-part anatomy, such as tentacles, mouths, or sense organs, and some have been reinterpreted as Ediacaran-like organisms, trace fossils, or abiotic pseudofossils [9], [15]–[17]. Medusoid fossils have been reported from the Mt. Simon-Wonewoc Sandstone (Upper Cambrian of Wisconsin, USA) and these appear to represent true medusozoans; however, because of their incomplete state of preservation, related to aspects of taphonomy [18], diagnostic characters of modern medusozoan classes were not preserved [19]. To date, the only Paleozoic fossils that definitively possess diagnostic medusozoan characters are from the Mazon Creek biota (Pennsylvanian of Illinois, USA) [20] and the Wea Shale (Pennsylvanian of Iowa, USA) [21].

Here we report exceptionally well-preserved medusozoan fossils from the Middle Cambrian Marjum Formation of Utah, USA. In contrast to the taphonomic expression of other Cambrian and Neoproterozoic medusoid fossils, which are impressions in coarse sediments, these new medusoid fossils exhibit Burgess Shale-type preservation [22] and are preserved as organic compressions on very fine-grained marine sediments. This allows the potential recovery of more detailed anatomical information, although it also entails that different specimens will show different features due to several preservational factors including how they are orientated to sediment bedding planes [23]. While the precise taphonomic processes responsible for preserving the remains of non-skeletonized soft-bodied animals, such as these medusozoans in the Marjum Formation, have not been studied in great detail, they have been considered for other soft-bodied remains derived from the slightly older Middle Cambrian Wheeler Formation of Utah by [24]. [24] have hypothesized that the mode of preservation in the two formations is likely similar, with factors associated with anoxic bottom waters that limit sediment permeability, bioturbation, and microbial-related decay facilitating the preservation of the exquisite detail of these soft-bodied remains. In this respect, soft-bodied preservation in the Marjum Formation is similar to what is known from the famous Burgess Shale [25]. Still, even with the level of detail preserved, we hesitate to definitively assign any of these fossils to a specific taxon because taphonomic factors can conspire to make particular features of specimens difficult to interpret and even modern jellyfish possess few diagnostic features. We do, however, discuss distinctive features exhibited by these specimens that indicate they share affinities with certain modern cnidarian clades.

## Materials and Methods

**Figure 1:**
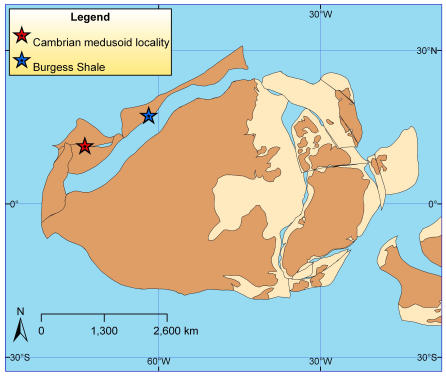
Paleogeographic reconstruction for the Middle Cambrian emphasizing the position of North America. Approximate positions of locality in Utah yielding fossil jellyfishes and the site of the famous Burgess Shale are marked as indicated. Map derived using [41].

**Figure 2:**
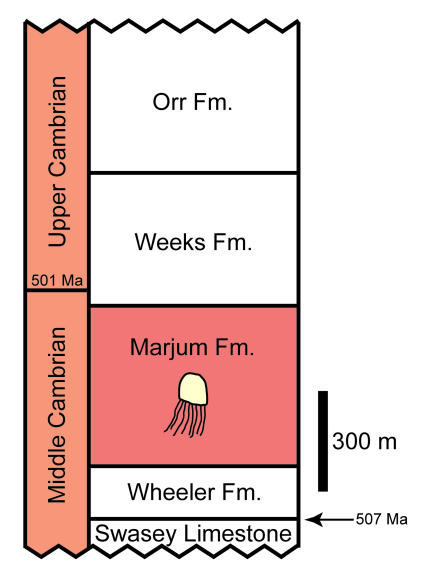
Stratigraphic column for Utah emphasizing Cambrian strata in the region where the new jellyfishes were collected. The new fossils come from an interval approximately 41–48 meters above the base of the Marjum Formation [26], [27]; approximate ages of some stratigraphic horizons are shown.

All fossil specimens were collected by co-authors SLH and RDJ and are from the Marjum Formation, Middle Cambrian, Utah, the Sponge Gully Locality, 39°16.143′N, 113°18.597′W. This locality, which has been described in detail previously [26]–[28] (Figs. 1, 2), yields a diverse biota of soft-bodied taxa and trilobites in inter-bedded mudstones and thin-bedded, fine-grained limestones. These rocks are inferred to represent deposition in a warm water environment on a gently sloping ramp below storm wave base [27], [28]. Specimens occur in thin (∼1 cm) fine-grained, dark grey mudstone beds that oscillate with thinner, more coarse-grained layers that weather to a reddish color.

## Results

**Figure 3:**
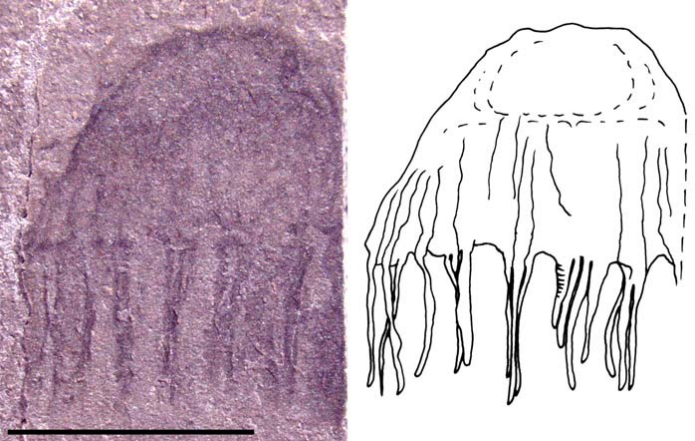
Photograph and interpretive drawing of Middle Cambrian cnidarian jellyfish in lateral view possibly referable to the family Narcomedusae, class Hydrozoa. Specimen UU07021.03, scale bar equals 5 mm.

**Figure 4:**
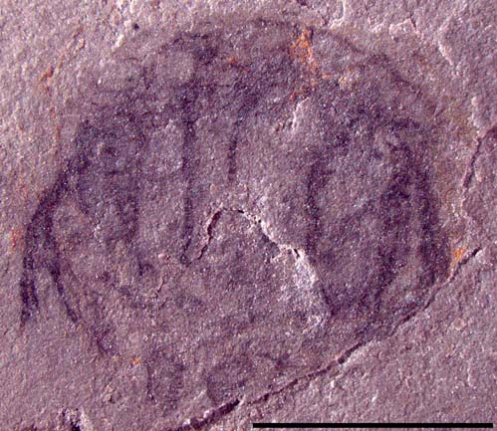
Photograph of Middle Cambrian cnidarian jellyfish in oblique subumbrellar view possibly referable to the family Narcomedusae, class Hydrozoa. Specimen UU07021.04, scale bar equals 5 mm.

Five specimens, UU [University of Utah] 07021.03 (Fig. 3), UU07021.04 (Fig. 4), UU07021.06, UU07021.07 and UU07021.08, appear to represent cnidarian medusozoans and have prominent umbrellas and trailing tentacles. In these specimens, the umbrella widths are greater than the umbrella heights, with a maximum width of 8.8 mm and a maximum height of 6.3 mm. Further, there are at least twelve solid tentacles, 3.7 mm in maximum length. The tentacles appear to arise on the exumbrella at some distance from the umbrellar margin. Due to the limited number of preserved characters it is difficult to determine how these specimens should be classified within Medusozoa. However, members of the hydrozoan family Narcomedusae do possess similar relative umbrella dimensions and have tentacle insertions above the umbrellar margin, suggesting that UU07021.03, UU07021.04, UU07021.06, UU07021.07 and UU07021.08 may possibly be narcomedusae.

**Figure 5:**
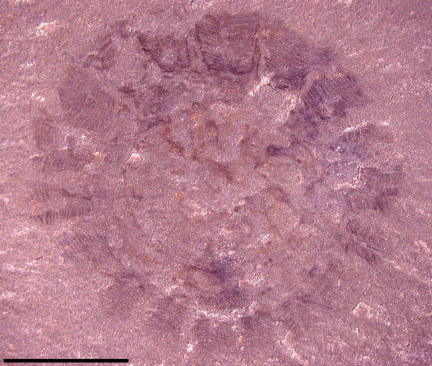
Photograph of Middle Cambrian cnidarian jellyfish in subumbrellar view possibly referable to the order Semaeostomeae, class Scyphozoa. Specimen UU07021.09, scale bar equals 5 mm.

**Figure 6 pone-0001121-g006:**
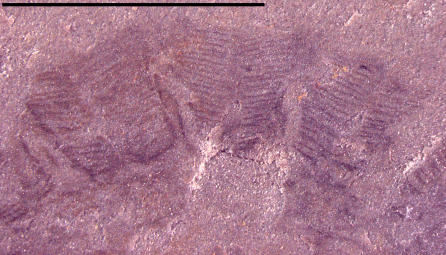
Close-up photograph showing structures interpreted as radially arranged coronal muscles. Specimen UU07021. 09.

**Figure 7 pone-0001121-g007:**
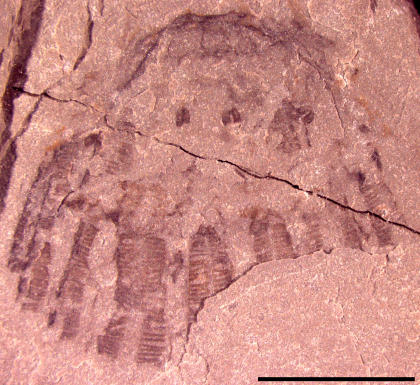
Photograph of Middle Cambrian cnidarian jellyfish in lateral view possibly referable to the order Semaeostomeae, class Scyphozoa. Specimen UU07021.10, scale bar equals 5 mm.

**Figure 8:**
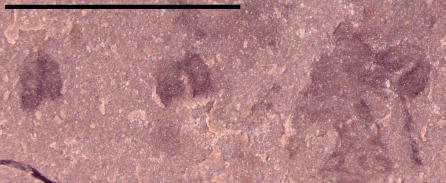
Close-up photograph showing structures interpreted as folded gonads. Specimen UU07021.10, scale bar equals 2.5 mm; these structures are visible in the upper middle part of Figure 10.

Two other specimens, UU07021.09 and UU07021.10 (Figs. 5–8), also appear to represent cnidarian medusozoans; however, these specimens display other characteristics distinct from the aforementioned material and instead share similarities with cnidarian jellyfish belonging to the class Scyphozoa. The specimen in Figure 5 is interpreted as a subumbrellar view of a jellyfish showing 18 pairs of radially arranged muscles; muscles are shown in a close-up view in Figure 6. These muscles are similar to the swimming musculature in an extant order of scyphozoans, the Semaeostomeae, which is arranged as a continuous coronal muscle interrupted by radial folds of the gastric cavity [29]. The outer margin of the umbrella in UU07021.09 is not preserved. UU07021.10 also appears to be a similar jellyfish but oriented laterally (Fig. 7). This specimen displays three horseshoe shaped structures (Fig. 8) that may be folded gonads, similar to those found in some extant semaeostomes [29]. The presence and arrangement of the swimming musculature and the possible presence of folded gonads are consistent with an assignment of these jellyfish to the scyphozoan order Semaeostomae, but given the uncertainties in preservation this assignment is made tentatively.

**Figure 9:**
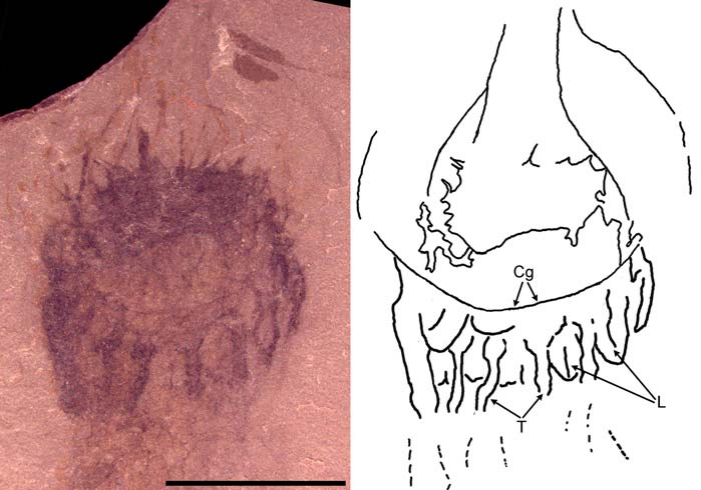
Photograph and interpretive drawing of Middle Cambrian cnidarian jellyfish in oblique lateral view possibly referable to the order Coronatae, class Scyphozoa. Specimen UU07021.05, scale bar equals 5 mm. Structure marked *Cg* in drawing interpreted as a coronal groove; structures marked *L* interpreted as lappets; structures marked *T* interpreted as tentacles.

Another cnidarian specimen, UU07021.05 (Fig. 9), shares similarities with a different order of scyphozoan jellyfish. For instance, it appears to have a dome-shaped umbrella, with a maximum height of 7.4 mm (obliquely oriented) and a maximum width of 7.1 mm, although the shape of the umbrella may be distorted because of the preservation of the specimen. There also appears to be a deep groove encircling the basal third of the exumbrellar surface which, due to its position on the umbrella relative to the tentacles, bears a resemblance to a coronal groove, a feature common to the scyphozoan order Coronatae. Distal to the umbrellar margin there appears to be at least six pointed lappets (approximately 1.1 mm in length from coronal groove to distal part of lappet) with the bases of the tentacles arising between successive lappets. The apparent shape of the bell, presence of probable pointed lappets, relative position of the tentacles to these lappets, and the presence of a coronal groove are all characteristics of the scyphozoan order Coronatae, but the lack of additional specimens makes it difficult to assign this specimen conclusively to that taxon. A Paleozoic coronate, *Octomedusa pieckorum,* has been described previously from the Mazon Creek biota (Pennsylvanian of Illinois, USA) [20] .

**Figure 10:**
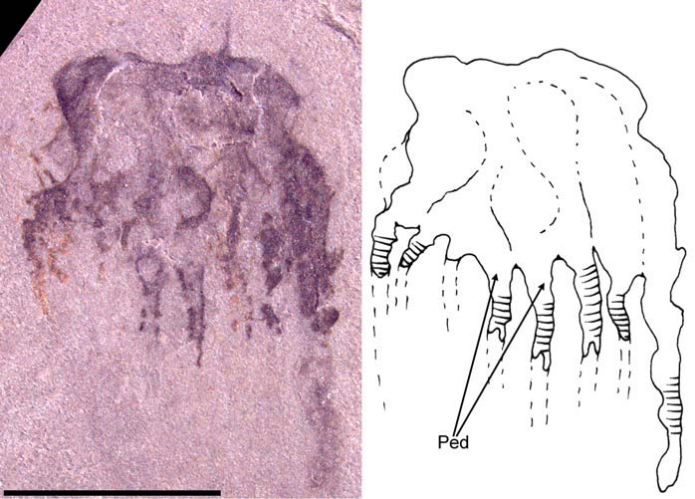
Photograph and interpretive drawing of Middle Cambrian cnidarian jellyfish in lateral view possibly referable to the class Cubozoa. Specimen UU07021.01, scale bar equals 5 mm. Structures marked *Ped* in drawing are interpreted as pedalia.

**Figure 11:**
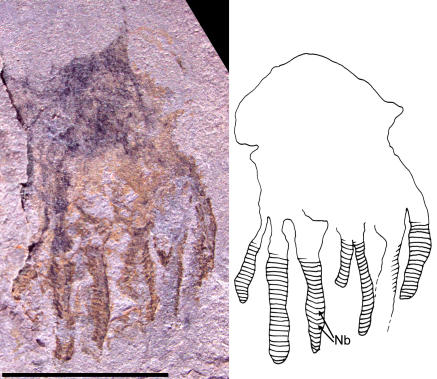
Photograph and interpretive drawing of Middle Cambrian cnidarian jellyfish in lateral view possibly referable to the class Cubozoa. Specimen UU07021.02, scale bar equals 5 mm. Structures marked *Nb* in drawing are interpreted as nematocyst batteries.

Finally, two other specimens, UU07021.01 and UU07021.02, appear distinct from the aforementioned material and have a box-shaped umbrella, with a maximum height of 6.3 mm, a maximum width of 7.2 mm, and a slightly concave apical region (Figs. 10, 11). There are thickenings at the proximal ends of the tentacles that are interpreted as pedalia. These pedalia are 2.8 mm in maximum height and 2.9 mm in maximum diameter. Each pedalium has one tentacle associated with it. The specimen in Figure 10 appears to display a central cluster of three pedalia and tentacles with two other clusters on either side. Tentacles have a maximum length of 5.2 mm and possess transverse lines, resembling chains of discs, that are interpreted as nematocyst batteries. Many cnidarians, including cubozoans, have nematocysts arranged in transverse rows or lines in the outer tissue of the tentacles and these form circumferential bands of nematocyst batteries [30]. These transverse nematocyst batteries are particularly evident in contracted tentacles [31]. It is true that fossil ctenophores also feature transverse lines that represent ctenes of comb rows [32]. However, the material illustrated here differs from ctenophore comb rows because there is a clear outer margin outlining the tentacles: this is absent in comb rows. In addition, the tentacles in the specimens reported here hang below the umbrellar margin (Figs. 10, 11). This is typical of cnidarian medusae and differs markedly from ctenophores, whose comb rows run along the entire oral/aboral axis of the body, e.g., see [33].

The square shape of the umbrella, the presence of pedalia, tentacles, and nematocyst batteries, as well as the arrangement of the tentacles around the umbrellar margin in specimens UU07021.01 and UU07021.02, are all characters of the class Cubozoa. We acknowledge that among the aforementioned traits umbrellar shape is most likely to be governed by taphonomic preservation; still, even setting this trait aside, in total these specimens show strongest affinities to this class of cnidarian jellyfish. The more than 30 extant species of Cubozoa [34] belong to two families: Carybdeidae, characterized by simple pedalia, each with one tentacle; and Chirodropidae, characterized by branched pedalia, each branch with its own tentacle. The only other reported Paleozoic cubozoan, *Anthracomedusa turnbulli* Johnson & Richardson, 1968, is from the Pennsylvanian Mazon Creek biota (Illinois, USA), and has been assigned to the Chirodropidae [20]. The new fossil material shows more traits in common with the Carybdeidae because the pedalia are simple and unbranched. However, given that taphonomic factors including incomplete preservation and orientation to bedding could influence the geometry of these structures, it is not possible to demonstrate conclusively that this material is referable to the Carybdeidae or even the Cubozoa.

## Discussion

These Middle Cambrian fossils are the oldest reported putative cnidarian jellyfish to display characteristics diagnostic of particular medusozoan taxa. If the preserved characters described herein are indeed consistent attributes of the fossil organisms, and not artifacts of taphonomy, then these Middle Cambrian fossils would be the oldest definitive crown-group jellyfish. Given the available character information, they also may comprise representatives of three separate classes of modern medusozoans: Cubozoa; Hydrozoa; and Scyphozoa. This suggests that an important aspect of modern marine pelagic ecosystems was in place shortly after the Cambrian radiation.

Extant medusozoans possess several complex characters. For example, the living cubozoan *Tripedalia cystophora* has sophisticated reproductive behavior that includes mate recognition and courtship, involving the indirect transfer of sperm through spermatophores [35]. Cubozoans also have complex eyes [36] and nervous systems [37]. The existence of our newly described fossil material may suggest that these complex traits could have evolved within the Cnidaria by the Middle Cambrian.

Finally, fossil anthozoans have recently been reported from the early Cambrian [38], while conulariids, which have a phylogenetic origin within the Medusozoa [39], are known from the Late Cambrian [40]. Taken in combination with the fossils discussed here, which may represent hydrozoans, scyphozoans, and cubozoans, it suggests that the modern cnidarian classes had evolved by the Cambrian. Further, some of these fossils share commonalities with modern cnidarian orders and families; this may indicate that a significant amount of diversification within the Cnidaria had also occurred by the Cambrian.
